# Effect of Punch Surface Grooves on Microformability of AA6063 Backward Microextrusion

**DOI:** 10.3390/mi12111299

**Published:** 2021-10-22

**Authors:** Tatsuya Funazuka, Kuniaki Dohda, Tomomi Shiratori, Ryo Hiramiya, Ikumu Watanabe

**Affiliations:** 1 Academic Assembly Faculty of Engineering, University of Toyama, Toyama 930-8555, Japan; shira@eng.u-toyama.ac.jp; 2 Department of Mechanical Engineering, Northwestern University, Evanston, IL 60201, USA; dohda.kuni@northwestern.edu; 3 Graduate School of Science and Engineering for Education, University of Toyama, Toyama 930-8555, Japan; m2071232@ems.u-toyama.ac.jp; 4 Research Center for Structural Materials, National Institute for Materials Science, Ibaraki 205-0047, Japan; WATANABE.Ikumu@nims.go.jp

**Keywords:** microextrusion, size effect, microtexture, grain size, aluminum alloy

## Abstract

In order to apply conventional forming processes at the micro scale, the size effects caused by material properties and frictional effects must be taken into account. In this research, the effects of tool surface properties such as punch surface grooves on microextrudability, assessed using extrusion force, shape of the extrusion, and Vickers hardness, were investigated using an AA6063 billet. Microscale grooves of 5 to 10 µm were fabricated on the punch surface. The extrusion force increased rapidly as the stroke progressed for all the grooves. Comparing the product geometries showed that, the smaller the groove size, the lower the adhesion and the longer the backward extrusion length. The results of material analysis using EBSD showed that a 5 µm groove depth punch improved the material flowability and uniformly introduced more strain. On the other hand, material flowability was reduced and strain was applied nonuniformly when a mirror-finish tool was used. Therefore, the tribology between the tool and the material was controlled by changing the surface properties of the punch to improve formability.

## 1. Introduction

In recent years, the improvement of production of microscale parts using plasticity processing technology has attracted attention in various fields, including medicine, electronics, and chemistry [1]. Among these technologies, microextrusion processing has attracted significant attention from industries as a microcomponent-forming processing technology. When conventional macroscale processing technologies such as extrusion are applied to the micro scale, problems arise in terms of repeatability and accuracy. Engel et al. clarified the effect of decreasing the product size on tribology using double-cup extrusion tests at the micro scale, and found that as the product size decreased, there were fewer pockets in the tool–billet contact area to hold lubricant. It was reported that as the product size decreased, the pockets that held lubricant in the tool–billet contact area decreased and the direct contact area increased, resulting in higher friction [2]. We also investigated the processing temperature suitable for microforming, and showed that stable forming is possible when processing at high temperatures where dislocation migration becomes active, and that the variation in product accuracy due to size effects is reduced [3].

In a series of studies on microextrusion [4,5,6,7], Cao et al. investigated the effects of microstructure and interface conditions such as grain size, shape, and orientation of the billet on processing. The deformation behaviors of different grain sizes in microextrusion were evaluated, and it was shown that the larger the grain size, the more easily the extruded part was bent due to nonuniform deformation, and that the difference in grain size affected the formability. In addition, the effectiveness of a hard coating to stabilize the friction during processing was investigated. Ngail et al. [8], Xu et al. [9] and Low et al. [10] designed a tool for ultrasonic microextrusion molding and investigated the effect of forming load and surface finish. To improve the micro-forming performance of metal material, ultrasonic vibration assisted for its superiorities in improving materials flow stress and reducing interfacial friction. Fu et al. [11,12] reported that the analysis of microforming can accurately predict the deformation behavior of the material by considering several influencing factors such as the flow pattern of the material, the state of the interface, and the flow stress curve. The author also reported the effects of microstructure, lubricant, and die coating on the forward–backward microextrusion technology of aluminum alloy [13,14,15]. The complex forward and backward material flow was evaluated by experiments and simulations, and it was proved that the backward extrusion was greatly affected by the friction between the die and the punch and microstructure.

In microforming, it is difficult to prepare surface properties suitable for the micro scale, and it is essential to find the optimum tool surface conditions to reduce friction and improve formability. In the case of microscale forming, the surface becomes relatively rough in relation to the processing scale. It has been shown that the roughness of the surface causes a large variation in friction and forming behavior [16,17]. In the field of micromachining, microtexturing has been applied to reduce the friction on the tool surface and to enlarge the lubrication pocket area, and the results have been satisfactory [18,19,20]. The microtexture is expected to reduce the tool contact area and stabilize the formability by retaining lubricant.

These studies have shown that in microextrusion, grain size control and tribology at the tool–material interface have a significant effect on formability parameters such as forming force and material flow. In this study, backward extrusion of microscale parts were investigated to realize microforming technology. The textured tool was used to reduce the tool contact area and to stabilize the formability by retaining lubricant. Microscale grooves were added to the punch surface and the effect of the punch surface properties was investigated. The effect of the grooves on the microextrudability was observed from the extrusion force, the shape of the extrusion, the amount of adhesion to the punches, and the microstructure analysis of the extrusion.

## 2. Materials and Methods

**Figure 1:**
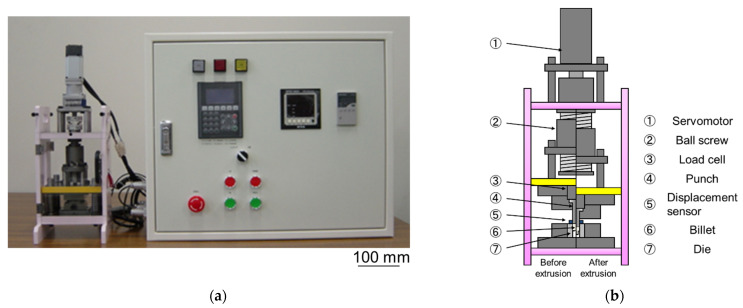
(**a**) Photograph of the experimental setup for microextrusion; and (**b**) schematic diagram of the extrusion apparatus.

Figure 1 shows the microextrusion device and the outline of the device. The microextruder is a servomotor-driven screw press. It controls the forming speed and position of a punch connected to the screw shaft via a load cell. The maximum output of the machine is 30 kN and the maximum stroke is 11.0 mm. The extrusion force and the punch stroke are fed back to the controller from the load cell and displacement meter, and the respective data can be obtained.

**Figure 2:**
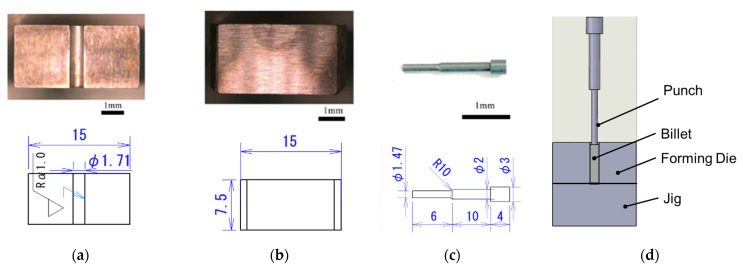
Dimensions of die and punch: (**a**) forming die, (**b**) jig, (**c**) punch, and (**d**) schematic view of backward microextrusion.

Figure 2 shows the outline of the die and punch. The die is divided at the center to take out the billet after extrusion. The die has a container inner diameter of φ 1.71 mm. The arithmetic mean roughness inside the container is Ra = 0.18 µm. The punch was selected for backward extrusion, and the diameter of the forming part is φ1.47 mm. The components shown in Figure 2a–c were assembled as shown in Figure 2d for the backward extrusion experiment.

**Figure 3:**
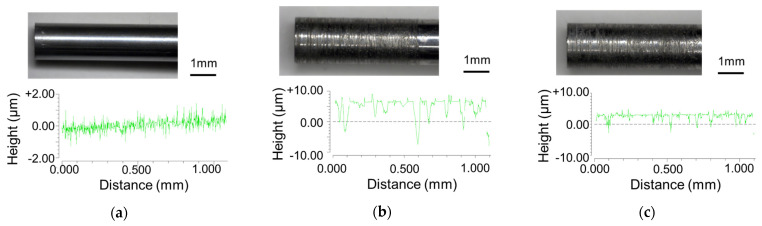
Grooved punches: (**a**) mirror surface punch; (**b**) grooved punch (depth 10 μm, about 10 grooves/mm); and (**c**) grooved punch (depth 5 μm, about 10 grooves/mm).

Figure 3 shows the punches used in this study. The grooves were measured using a noncontact three-dimensional roughness measurement system. Figure 3a shows a punch with a ground surface and a mirror finish. Figure 3b shows a punch with a groove of 10 µm made by using 140 grit abrasive paper on the punch shown in Figure 3a. Figure 3c shows a punch with grooves of about 5 µm in depth and pitch of about 100 µm, made by using 400 grit abrasive paper on the punch shown in Figure 3a.

The test billets were cut from A6063 aluminum alloy φ 1.70 mm round wire and finished to a length of 4.0 mm. Table 1 shows the shape dimensions, grain size, mechanical properties, and microstructure of the billets. The average grain size of the billets was 23.3 µm. The relationship between the true stress *σ* and the true strain *ε* of the billet can be expressed by the hardening Equation (1), which is the power of the plasticity factor *F* [MPa] and the work hardening index *n*. Since the microscale is greatly affected by the micro structure, it is necessary to evaluate the mechanical properties according to its dimensions. The true stress *σ* and the true strain *ε* were derived from microcompression tests using billets with a φ 1.70 mm and a length of 2.5 mm [13,14]. The compression ratio was set to 80% and the compression speed to 0.1 mm/s. The plasticity factor *F* and work hardening index *n* were derived through this microcompression test.

*σ* = *F*
*ε*
^*n*^ (1)

The extrusion conditions were set at room temperature with a ram speed of 0.1 mm/s and a ram stroke of 1.5 mm. In this experiment, the extrusion test was repeated four times with each billet to confirm the repeatability. An electron probe micro analyzer (EPMA) was used to evaluate the adhesion to the punches. Electron back-scattered diffraction pattern (EBSD) and micro Vickers hardness tests were used for product microstructure analysis. The orientation distribution and grain size of the grains were analyzed using the EBSD method in order to measure the effect on the material when using the tool with reduced friction by the groove punch. 800 μm × 0.15 mm from the tip of the extrusion was measured. The sample was polished to a mirror finish using an abrasive, and residual stress on the surface was removed by ion milling. The measurement conditions were an acceleration voltage of 20 kV and irradiation current of 13 nA. For the measurement at the tip of the extrusion, step size 0.5 µm was used, and for the local measurement, step size 0.1 µm was used.

## 3. Experimental Results and Discussion

### 3.1. Extrusion Force–Ram Stroke Diagram and Metal Flow during Backward Microextrusion

**Figure 4:**
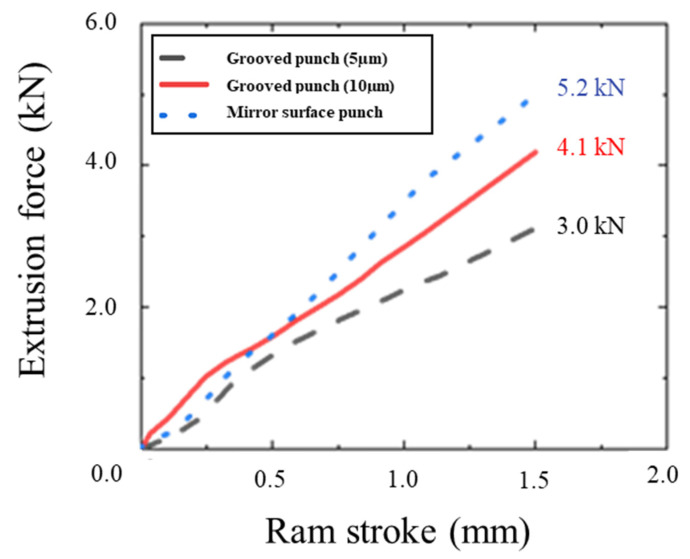
Extrusion force–ram stroke curve in each punch.

Figure 4 shows the extrusion force–stroke diagram when using a mirror punch, a 10 µm groove punch, and a 5 µm groove punch. The extrusion force was 5.2 kN with the mirror surface punch, 4.1 kN with the 10 µm groove punch, and 3.0 kN with the 5 µm groove punch. This section may be divided by subheadings. It should provide a concise and precise description of the experimental results, their interpretation, as well as the experimental conclusions that can be drawn.

**Figure 5:**
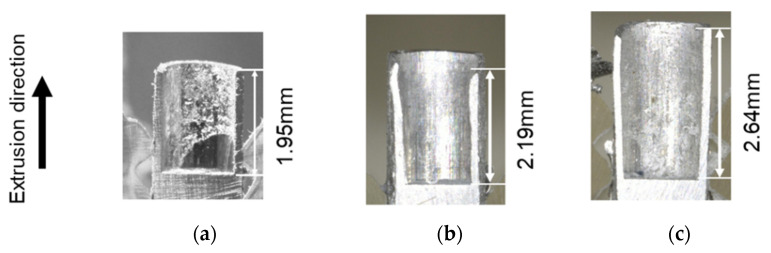
Longitudinal section cross-sectional images of the extrusion: (**a**) mirror surface punch; (**b**) grooved punch (depth 10 μm); and (**c**) grooved punch (depth 5 μm).

Figure 5 shows the longitudinal section cross-sectional shape of product after backward microextrusion for each punch and the backward extrusion length (lb) at a ram stroke of 1.5 mm. The backward extrusion lengths (lb) were 1.95 mm, 2.19 mm, and 2.63 mm for the mirror, 10 µm groove, and 10 µm groove punches, respectively, indicating that the microscale texture on the punches increased the backward extrusion length by reducing the true contact area between the billet and the tool. This indicates that the addition of texture to the tool reduced the contact area and thus the friction. It is thought that this will result in smoother plastic flow and a longer backward extrusion length. The reason for the shorter backward extrusion length observed when using the 10 µm groove punch than the 5 µm groove punch is that there are two flows: one in the backward extrusion direction and one that enters the groove of the tool. By reducing the texture depth, the aluminum adhesion of the punch was broken up and the friction was reduced. As a result, the plastic flow became smoother and the backward extrusion length became longer. Therefore, it is considered that a friction reduction effect can be obtained by reducing the texture depth in microscale forming.

### 3.2. Evaluation of Adhesion to Punch

**Figure 6:**
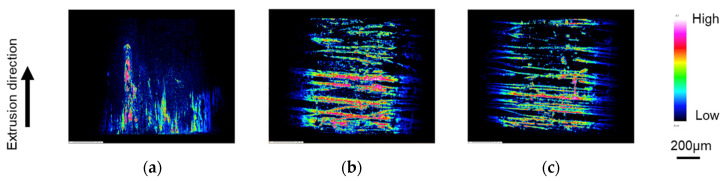
Evaluation of adhesion to punch by EPMA: (**a**) mirror surface punch; (**b**) grooved punch (depth 10 μm); and (**c**) grooved punch (depth 5 μm).

Figure 6 shows the amount of adhesion on the surface of the punches after machining with the different surface properties of the mirror punch, 10 µm grooved punch, and 5 µm grooved punch. EPMA was used to analyze the punch surfaces.

The experimental results show that the aluminum entered the circumferential grooves and the adhesion was divided. In addition, when the amount of adhesion was compared between the two punches with different sizes of textured grooves on the tool surface, the amount of condensation was smaller on the 5 µm grooved punch than on the 10 µm grooved punch. This is thought to be due to the fact that the smaller the groove size, the smaller the pockets into which the material flows, and the finer the deposition. It is also considered that the frictional force is reduced by breaking up the adhesion, and the force is reduced.

### 3.3. Microstructure Analysis of the Extrusion

**Figure 7:**
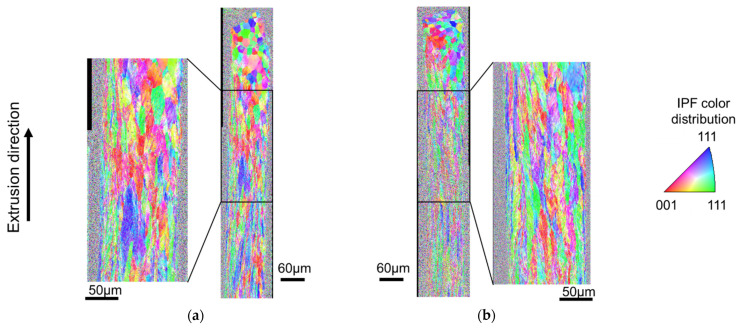
IPF map of the extrusion by EBSD: (**a**) mirror surface punch; and (**b**) grooved punch (depth 5 μm).

**Figure 8:**
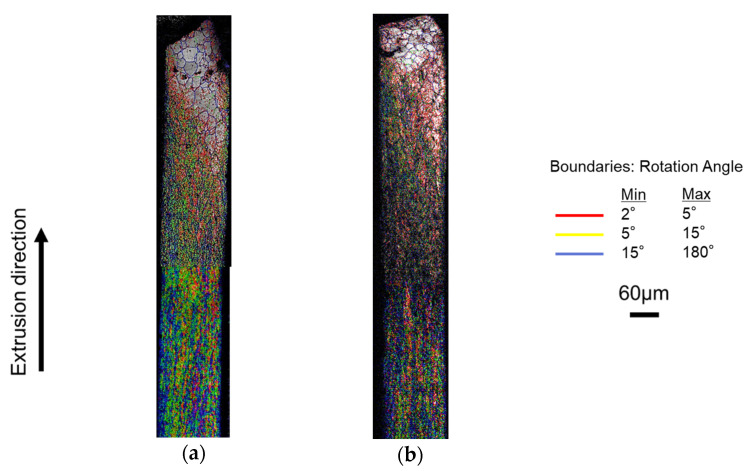
IQ map of the extrusion by EBSD: (**a**) mirror surface punch; and (**b**) grooved punch (depth 5 μm).

Figure 7 shows the results of Inverse Pole Figure (IPF) map when using a mirror punch and a 5 µm groove punch. The IPF map is defined by the crystal plane and is a measurement method for determining crystal orientation by color. Figure 8 shows the results of the Image Quality (IQ) map when using a mirror punch and a 5 µm groove punch. The IQ map is colored by a low angle grain boundary of 2–5°, a middle angle grain boundary of 5–15°, and a high angle grain boundary of 15–180°. In the case of the mirror punch, the grain size at the tip of the extrudate was not sheared and flowed out as it was. From the shape of the high angle grain boundary and IPF map, the material was sheared in the longitudinal direction and the shape of grain was stretched at the end of the extrusion. Near the center of the extrusion, the grain size before processing was slightly collapsed into an oval shape. The color of the crystal orientation was uneven, indicating that the crystal state was changing due to the shear force in the extrusion direction. Near the rear end of the extrudate, the crystal was elongated in the longitudinal direction. Compared to the mirror punch, the 5 µm grooved punch had a longer grain size and more noise in the longitudinal direction. It is thought that the greater strain of the 5 µm grooved punch compared to the mirror punch may have increased the noise in the measurement.

**Figure 9:**
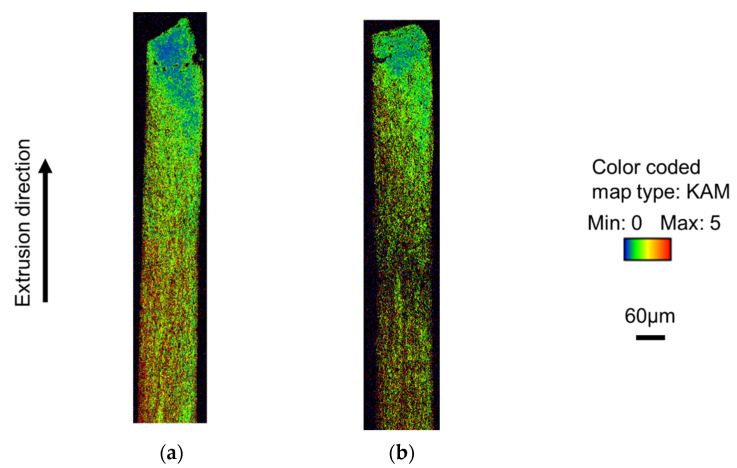
KAM map of the extrusion by EBSD: (**a**) mirror surface punch; and (**b**) grooved punch (depth 5 μm).

Figure 9 shows the results of the Kernel Average Misorientation (KAM) map, which is a measurement method used to quantitatively evaluate the residual strain inside a measured sample based on the orientation difference information. In the mirror punch, the tip of the material was dominated by green and yellow with an orientation difference of 1 to 2°, while the rear end of the material was dominated by red and yellow with an orientation difference of 3 to 5°. In contrast, the 5 µm grooved punch showed a uniform strain of more than 3 to 5° from the middle to the end of the material. This suggests that the 5 µm grooved punch reduced the friction and facilitated the material flow, resulting in uniform strain and accelerated machining progress. On the other hand, for the mirror punch, the friction was higher and the material flow was more difficult, resulting in nonuniform strain accumulation.

**Figure 10:**
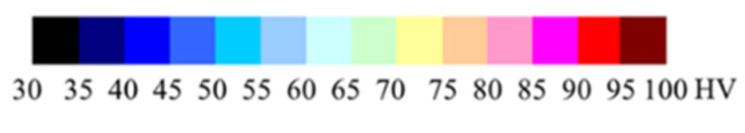

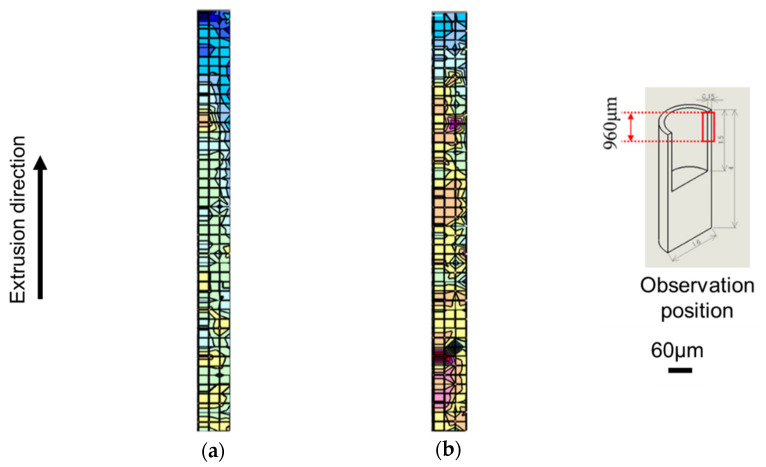
Distribution of the Vickers hardness of the extrusion: (**a**) mirror surface punch; and (**b**) grooved punch (depth 5 μm).

Although KAM mapping is a useful measurement method for processes that apply local strain such as shearing, it has been pointed out that the internal strain distribution is affected by the grinding state for processes that apply large deformation overall, such as forging. Therefore, the hardness was measured using a Vickers hardness tester to support the KAM mapping. Figure 10 shows the Vickers hardness distribution in the longitudinal section of the product when a mirror punch and a 5 µm grooved punch were used. It can be seen that the hardness of the mirror punch was about 75 HV at the backward end of the material, while the hardness of the 5 µm grooved punch was 70–90 HV. This indicates that the 5 µm grooved punches were more work-hardened due to strain. This result suggests that the noise observed for the 5 µm grooved punches in the IPF and KAM mapping was not only noise due to the difference in the surface condition of the polish, but was also likely to be noise caused by a large number of dislocations. In addition, the KAM map showed that there was a lot of strain on the left side of the material, the surface in contact with the punch. In the present Vickers hardness test, the left side of the surface was work-hardened more than the right side for both punches, suggesting the same tendency as was observed in the KAM map.

In conclusion, microsized texturing was more effective in terms of formability and friction reduction stabilization in microforming. By reducing the true contact area between the billet and the tool, the material flowability was improved and the backward extrusion length was increased. This suggests that adding texture to a tool can reduce the contact area and reduce friction.

## 4. Conclusions

1. The extrusion force–stroke diagram of the backward microextrusion process increased gradually with increasing stroke. The extrusion force was reduced by adding microscale grooves.
2. In the shape of the extrusion, the backward extrusion length became shorter under the condition of high extrusion force. In the case of punches with small groove depths, the material flowability was improved and the backward extrusion length became longer.
3. From the evaluation of adhesion on the punch surface by EPMA, it became possible to break up the adhesion size to a state suitable for the processing scale by adding microscale grooves to the punch surface. Therefore, the microgrooves had a good friction reduction effect and good formability in microforming.
4. According to the IPF map and KAM map by EBSD and the Vickers hardness distribution, the microscale grooves on the punch improved the material flowability, and the strain distribution inside the extrusion became more uniform.

In future research, we will investigate the effect of surface properties at the nano scale.

## Figures and Tables

**Table 1:** Dimension and properties of AA6063 billet.

Item	Value and Figure
Billet Shape	φ 1.70 × 4 (mm)
Vickers hardness	33.2 (HV)
*F*	169.0 (MPa)
*n*	0.29
Microstructure	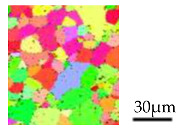
Grain distribution	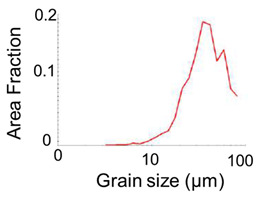
Average grain size	23.3 (μm)
